# Tryptophan Residues’ Incorporation Modulates Ferritin Thermal Stability and Hydrophobicity

**DOI:** 10.3390/cimb48070710

**Published:** 2026-07-11

**Authors:** Luisa Affatigato, Sara Anselmo, Anna Fricano, Giuseppe Sancataldo, Mariano Licciardi, Alessio Incocciati, Alessandra Bonamore, Alberto Macone, Alberto Boffi, Valeria Militello

**Affiliations:** 1 Department of Physics and Chemistry—Emilio Segrè, University of Palermo, 90128 Palermo, Italy; luisa.affatigato@unipa.it (L.A.); sara.anselmo@unipa.it (S.A.); anna.fricano@unipa.it (A.F.); valeria.militello@unipa.it (V.M.); 2 Department of Biological, Chemical and Pharmaceutical Sciences and Technologies (STEBICEF), University of Palermo, 90123 Palermo, Italy; mariano.licciardi@unipa.it; 3 Department of Biochemical Sciences—A. Rossi Fanelli, Sapienza University, 00185 Rome, Italy; alessio.incocciati@uniroma1.it (A.I.); alessandra.bonamore@uniroma1.it (A.B.); alberto.macone@uniroma1.it (A.M.); alberto.boffi@uniroma1.it (A.B.)

**Keywords:** ferritin, tryptophan, protein engineering, ANS fluorescence, thermal stability, hydrophobicity

## Abstract

Ferritin, a physiological iron-storage protein, has emerged as a highly attractive platform for drug delivery owing to its biocompatibility, structural robustness, and intrinsic ability to encapsulate and protect therapeutic cargo within its hollow nanocage. Building upon previous studies that established the baseline characteristics of engineered ferritin mutants in comparison to the wild-type protein, this work specifically investigates and directly compares the thermal stability profiles of two distinct mutated variants. These variants of human H-chain ferritin, obtained through targeted site-directed mutagenesis, feature either four or six tryptophan residues per subunit, strategically positioned toward the inner cavity of the protein shell. These modifications were intended to enhance hydrophobic interactions with guest molecules while preserving the native quaternary architecture. Temperature-dependent changes in surface hydrophobicity and solvent accessibility were probed using the environment-sensitive fluorescent dye ANS, enabling a comparative assessment of the conformational behavior of the two mutants. Overall, this study highlights how targeted modulation of the internal cavity composition of ferritin can tune both its physicochemical properties and stability, providing insights relevant for the rational design of ferritin-based nanoplatforms for biomedical applications.

## 1. Introduction

Nanoparticle-based drug delivery systems have emerged as a powerful strategy to overcome the major limitations associated with conventional pharmacological treatments, including poor solubility, rapid systemic clearance, off-target toxicity, and suboptimal biodistribution [1,2,3]. By engineering materials at the nanoscale (1–100 nm), it is possible to modulate pharmacokinetics, enhance drug stability, and improve tissue-specific accumulation through both passive and active targeting mechanisms.

Nanocarrier platforms can be broadly categorized into synthetic systems (e.g., liposomes, polymeric nanoparticles, dendrimers, inorganic nanoparticles) [4,5,6] and biomolecule-based systems, including protein nanocages. While synthetic nanoparticles offer tunable physicochemical properties, concerns regarding long-term biocompatibility, biodegradability, and immunogenicity remain relevant; protein-based nanocarriers, in contrast, provide intrinsic biodegradability, structural precision at the atomic level, and the possibility of genetic engineering for fine control over size, surface chemistry, and internal cavity properties.

Among protein nanocages, ferritin has attracted considerable attention due to its well-defined architecture, reversible self-assembly, and capacity for cargo encapsulation [7,8,9,10]. Human H-ferritin (HFt) is a self-assembling 24-mer protein nanocage (outer diameter ~12 nm; inner cavity ~8 nm) widely investigated as a biomimetic nanocarrier for drug delivery applications. Its intrinsic biocompatibility, precise supramolecular architecture, pH-dependent reversible disassembly, and natural recognition of transferrin receptor 1 (TfR1), which is frequently overexpressed in cancer cells, make HFt a promising platform for targeted nanomedicine applications [11,12]. Conventional drug-loading strategies rely either on pH-induced disassembly/reassembly or on diffusion through the three-fold channels of the cage [13]. Several studies have also used chaotropic agents such as urea and guanidine hydrochloride to induce transient expansion of ferritin channels, thus facilitating the diffusion of guest molecules into the nanocage without requiring complete disassembly [12,14,15,16].

However, the encapsulation efficiency of highly hydrophobic molecules remains limited due to the predominantly hydrophilic nature of the inner cavity [17].

To address this intrinsic limitation, several protein engineering strategies have been explored to modulate the physicochemical properties of the internal cavity. Early approaches focused on the insertion of short peptide sequences to introduce additional interaction motifs within the nanocage lumen [18]. Although these strategies demonstrated the feasibility of tuning cargo affinity by increasing the overall hydrophobic character of the internal cavity, they also introduced steric constraints that partially reduced the available internal volume, thereby limiting cargo penetration and overall loading capacity. More recently, a shift toward minimal but highly effective mutagenesis strategies has been proposed. In particular, the introduction of hydrophobic aromatic residues, especially tryptophan, has emerged as a powerful alternative [19,20]. Tryptophan residues, when strategically positioned toward the internal cavity, enhance non-covalent interactions such as π–π stacking and hydrophobic effects with aromatic and poorly soluble drug molecules, without significantly compromising the structural integrity or volume of the nanocage. This approach preserves the native architecture of ferritin while creating a more favorable microenvironment for the encapsulation of hydrophobic therapeutics, thereby improving loading efficiency and potentially modulating release kinetics [9].

In this context, hydrophobicity-enhanced variants of human H-ferritin, namely HFt-W4 and HFt-W6, were developed through site-directed mutagenesis by substituting selected internal residues with tryptophan [9].

HFt-W4 carries four internal mutations (H58W, H61W, H129W, H152W), strategically positioned to line the inner cavity without perturbing inter-subunit interfaces. HFt-W6 includes two additional substitutions (Y138W, F171W), further enhancing lumen hydrophobicity.

A previous study by Incocciati et al. (2023) [9] evaluated the stability of these engineered ferritin variants, as well as their ability to undergo reversible pH-dependent disassembly and reassembly, compared to wild-type (unmodified) ferritin. Notably, drug-loading experiments with hydrophobic compounds such as ellipticine have demonstrated that increased internal hydrophobicity significantly enhances molecular uptake, with HFt-W4 showing a markedly higher encapsulation capacity compared to wild-type ferritin while preserving its structural integrity.

Building upon these findings, the present study shifts focus toward a comparative analysis between the two mutated systems under thermal stress, a parameter of crucial relevance for technological applications. Investigating their behavior as a function of temperature is highly rationalized by drug-delivery kinetics; previous studies on ferritin platforms have demonstrated that elevated temperatures can significantly increase the drug-loading efficiency [17,21,22,23,24] by inducing structural fluctuations that modulate the gating, size, and accessibility of the ferritin channels.

At the same time, temperature is known to have a significant impact on protein structural stability, potentially inducing partial unfolding, misfolding, and aggregation phenomena. Therefore, this approach aims to elucidate how increasing temperature simultaneously affects internal cavity properties and the overall conformational integrity of the protein nanocage [14,25].

To this end, spectroscopic techniques, in combination with the environment-sensitive fluorescent probe 8-anilino-1-naphthalenesulfonic acid (ANS), were employed to monitor temperature-induced structural rearrangements and changes in the internal hydrophobic landscape of the nanocage. The solvatochromic dye ANS was used as a reporter of hydrophobic surface exposure and cavity accessibility [26,27]. Owing to its strong fluorescence enhancement in non-polar environments, ANS provides a sensitive readout of variations in the lumen microenvironment and of potential temperature-dependent exposure of hydrophobic patches.

Complementary spectroscopic measurements further provided insight into the global structural stability and unfolding behavior of the engineered ferritin variants upon heating. Overall, the integration of these approaches enables us to link thermal stress to both macroscopic conformational stability and local alterations in cavity hydrophobicity, offering a detailed picture of how internal tryptophan engineering modulates the structural robustness and environmental responsiveness of ferritin nanocages.

## 2. Materials and Methods

### 2.1. Chemicals and Reagents

Sodium phosphate monobasic (55,011), sodium phosphate dibasic (71,640), and 1-anilino naphthalene-8-sulfonate (A-1028) were purchased from Sigma-Aldrich (St. Louis, MO, USA). Sodium chloride (71,378) was purchased from Fluka (Buchs, Switzerland).

### 2.2. Protein Expression and Purification

HFt-W4 and HFt-W6 mutants were generated from synthetic genes optimized for expression in *Escherichia coli* and cloned into the pET22b vector. Recombinant proteins were expressed and purified following the procedure previously described by Incocciati et al. [9]. Briefly, purification involved ammonium sulfate fractionation, heat treatment to remove thermolabile contaminants, and size-exclusion chromatography. Protein purity and assembly state were verified by SDS-PAGE and high-performance size-exclusion chromatography, while concentrations were determined spectrophotometrically at 280 nm using construct-specific extinction coefficients.

### 2.3. Steady-State Fluorescence Emission Spectra

Fluorescence measurements were acquired using a Jasco FP-8500 spectrofluorometer (Jasco, Tokyo, Japan) equipped with a Jasco ETC-815 Peltier as a temperature controller in 1 cm path length quartz cuvettes.

### 2.4. Fluorescence and Rayleigh Scattering

Intrinsic luminescence emission spectra of 5 μM HFt-W4 and HFt-W6 in 20 mM sodium phosphate buffer (pH 7.4) supplemented with 150 mM NaCl were recorded at 0.5 nm wavelength intervals with excitation and emission bandwidths of 2.5 nm, a scan speed of 100 nm/min, and an integration time of 1 s. For kinetic experiments, after thermal equilibration, intrinsic fluorescence emission spectra were collected upon excitation at 280 nm over the 270–550 nm range every three minutes during heating from 25 °C to 80 °C.

Simultaneously, Rayleigh scattering intensity at 90° was measured as the maximum intensity of the elastic peak of excitation light at 280 nm.

### 2.5. ANS Fluorescence Measurements

1-anilino naphthalene-8-sulfonate (ANS) emission spectra were acquired in the range 370 nm–650 nm using λ_exc_ = 380 nm with an excitation bandwidth of 5 nm, an emission bandwidth of 5 nm, a response time of 1 s, a data interval of 0.5 nm, and a scan speed of 100 nm/min. The ANS concentration was 30 μM.

Spectra were acquired at each temperature between 25 °C and 80 °C. All spectra were corrected for background fluorescence by subtracting the corresponding buffer spectrum acquired with identical parameters.

ANS fluorescence spectra were analyzed in terms of the Generalized Polarization (GP) function, calculated using the following formula adapted from Parasassi et al. [28]:

$$GP=\frac{I_{480}-I_{525}}{I_{480}+I_{525}}$$

where *I*_480_ and *I*_525_ are the fluorescence intensities at 480 nm and 525 nm, respectively. An increase in GP values indicates a spectral blue shift of the emission band, whereas a decrease corresponds to a red shift.

### 2.6. Circular Dichroism

Circular dichroism (CD) spectra of 5 µM HFt-W4 and HFt-W6 were recorded using a J-715 spectropolarimeter (Jasco, Tokyo, Japan) equipped with a Jasco PCT 348WI temperature controller. Measurements were performed in the far-UV region (190–260 nm) using quartz cuvettes with a 0.1 mm path length. Spectra for both samples were acquired before and after incubation at 75 °C for 5 h.

Each spectrum represents the average of ten accumulations, collected with a 0.5 nm data interval, a bandwidth of 1 nm, and a scan speed of 50 nm/min. All spectra were acquired at room temperature.

## 3. Results and Discussion

**Figure 1:**
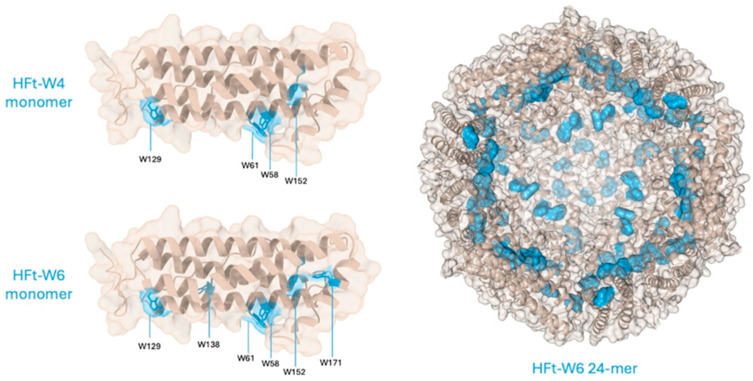
Structural mapping of engineered tryptophan residues in HFt variants. On the left, cartoon and transparent surface representations of the HFt-W4 monomer are shown, highlighting the spatial arrangement of the four selected tryptophan residues (W58, W61, W129, and W152), shown in blue (sticks and surface), and the HFt-W6 monomer, showing the inclusion of two additional tryptophans (W138 and W171). On the right, the quaternary structure of the assembled HFt-W6 24-mer nanocage is presented. The topological mapping highlights the predominant localization of the engineered tryptophan network (blue) facing the internal luminal cavity of the protein shell.

HFt-W4 and HFt-W6 are engineered variants of human H-chain ferritin (HFt) designed to introduce defined surface functionalities while preserving the native quaternary architecture. Specifically, selected histidine, phenylalanine, and tyrosine residues whose side chains are oriented toward the internal cavity were replaced with tryptophan residues in each subunit, resulting in the incorporation of either four or six substitutions per subunit, as reported in Figure 1.

These substitutions are designed to maintain the integrity of the α-helical fold and the ferroxidase center [9], while providing controlled aromatic patches for spectroscopic tracking and/or intermolecular interactions. Given the 24-subunit architecture of ferritin, the HFt-W4 and HFt-W6 mutants incorporate an additional 96 and 144 tryptophan residues per assembled nanocage, respectively, relative to the wild-type protein. This increased tryptophan content enables direct spectroscopic monitoring through intrinsic fluorescence measurements.

We exploited this feature to investigate the stability and aggregation propensity of the two ferritin mutants. Although the effects of pH on ferritin structure and cage stability have been studied in previous works [9], relatively little attention has been paid to the role of temperature. Temperature is a particularly relevant parameter, as it directly influences protein dynamics, conformational stability, and intermolecular interactions, all of which can, in turn, affect the integrity of the ferritin nanocage and its propensity for aggregation. Furthermore, thermal stimuli have been used to modulate ferritin permeability and facilitate molecule encapsulation in nanotechnology and drug delivery applications [17,22,23,24,25]. Therefore, studying the thermal response of engineered variants provides valuable insights into their structural stability and potential applicability. In particular, the aggregation process was monitored via Rayleigh scattering, which measures the intensity of elastically scattered light and is widely utilized as a sensitive indicator of particle formation and macromolecular assembly [29]. An increase in Rayleigh scattering intensity directly reflects the progressive formation of higher-order structures over time.

**Figure 2:**
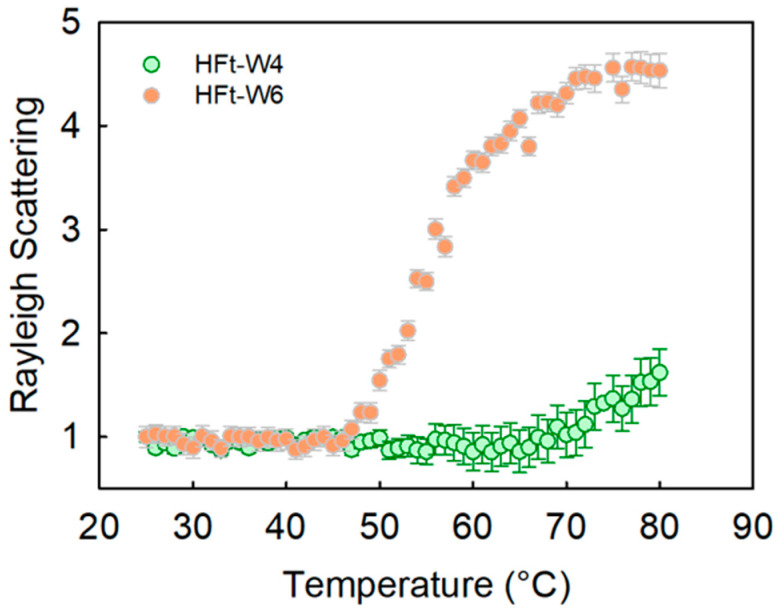
Rayleigh scattering intensity as a function of temperature for the two engineered ferritin variants, HFt-W4 (green) and HFt-W6 (orange), at a final protein concentration of 5 μM in 20 mM sodium phosphate buffer (pH 7.4) supplemented with 150 mM NaCl. The data were normalized to the first measurement (25 °C). Error bars represent the standard deviation.

Figure 2 reports the variation in Rayleigh scattering intensity during an upward temperature scan (12 °C/h) for 5 mM HFt-W4 (orange) and HFt-W6 (green). From the profiles, it is immediately evident that the two mutants exhibit markedly different aggregation behavior. In both cases, scattering remains essentially constant up to a threshold temperature, defining a lag phase in which the system is kinetically stable and no detectable aggregation occurs. Above this regime, a sharp increase in scattering marks the onset of aggregation.

Notably, HFt-W6 shows an earlier transition, with aggregation starting at approximately 48 °C, whereas HFt-W4 remains stable up to significantly higher temperatures (~70 °C). This clear shift indicates reduced thermal stability and a higher aggregation propensity for HFt-W6 compared to HFt-W4. The shorter lag phase observed for HFt-W6 further supports this interpretation, suggesting a lower energetic barrier for nucleation of aggregation-prone species and a faster transition toward the aggregated state once destabilization begins [30].

These findings are consistent with the work of Incocciati et al. [9], who, using pH-jump experiments, reported an incorrect quaternary assembly of HFt-W6 during the disassembly and reassembly process, further highlighting the reduced structural stability of this mutant.

To investigate the role of hydrophobic interactions in protein stability, we employed 8-anilinonaphthalene-1-sulfonic acid (ANS). This fluorescent probe is widely established in the literature as a sensitive reporter of protein surface hydrophobicity and conformational changes [26,27], including under temperature-induced unfolding conditions [31,32,33]. ANS is virtually not fluorescent in aqueous solutions but selectively binds to exposed hydrophobic regions of proteins, resulting in a dramatic increase in fluorescence intensity and a characteristic blue-shift of its emission spectrum [26,27]. Here, ANS was utilized to monitor variations in solvent-exposed apolar surfaces associated with the thermal rearrangements of the 24-mer assembly. The resulting emission spectra were analyzed in terms of the Generalized Polarization (GP) parameter (see Section 2.5 for details), a metric commonly used with solvatochromic probes to report on changes in local polarity and environmental dynamics [26,28,34]. Although GP is also sensitive to microenvironmental viscosity and probe rotational dynamics, it serves in this context as a reliable indicator to monitor protein structural reorganization and the consequent exposure of hydrophobic patches to ANS under thermal stress. In this framework, negative GP values indicate a more polar, solvent-exposed environment, whereas positive values reflect a more hydrophobic context. This latter condition is typically associated with an enhanced exposure of non-polar residues, such as tryptophan, to the probe, suggesting a local rearrangement of the native packing and a higher degree of protein instability.

**Figure 3:**
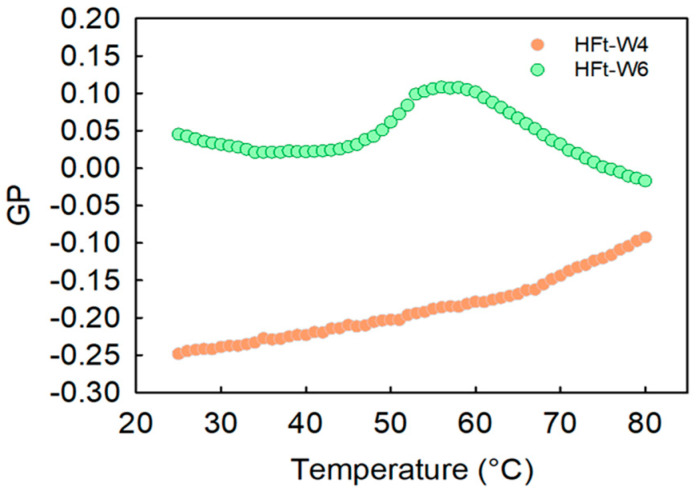
Temperature-dependent fluorescence analysis of ANS (30 μM), expressed in terms of the Generalized Polarization (GP) parameter, in the presence of 5 μM HFt-W4 and HFt-W6 in 20 mM sodium phosphate buffer (pH 7.4) with the addition of 150 mM NaCl. The plots show the evolution of GP as a function of temperature, providing insight into changes in the local polarity and the exposure of hydrophobic regions during the aggregation process. Variations in GP reflect the dynamic rearrangement of protein structure, with decreases indicating increased exposure of hydrophobic patches and increases corresponding to more solvent-exposed, polar environments. These measurements were repeated in triplicate.

As reported in Figure 3, the two mutants already exhibit markedly different GP values at the initial temperature, suggesting distinct starting conformational states. In particular, W4 exhibits a negative GP value, whereas W6 displays a positive GP value. These opposite GP values indicate distinct local environments for the introduced tryptophan residues. The negative GP observed for W4 suggests that a significant fraction of hydrophobic surfaces is already accessible to the solvent, consistent with a less compact or more dynamically fluctuating structure. In contrast, the positive GP value observed for W6 indicates that the introduced tryptophan residues are predominantly located in structurally compact regions, where they are shielded from the solvent and not readily accessible to ANS.

This is in agreement with previous findings showing a significant increase in ANS fluorescence for W6 upon chemical stress, which suggested an increased exposure of internal hydrophobic residues potentially related to an incorrect quaternary assembly [9]. Upon increasing temperature, W4 shows a progressive increase in GP. On one hand, this behavior can be attributed to a gradual unfolding process leading to the exposure of previously buried residues and increased solvent accessibility, suggesting a relatively cooperative and continuous structural transition without a pronounced accumulation of intermediate states. Alternatively, this GP trend can be ascribed to thermally induced structural fluctuations that enhance the overall accessibility of the nanocage; this process could facilitate, in accordance with the literature [17,21,23,24], the penetration of the probe through the ferritin channels into the lumen, promoting its interaction with internal hydrophobic regions. Conversely, W6 exhibits a non-monotonic GP trend, characterized by an initial decrease, followed by an increase and a subsequent decline at higher temperatures. The initial decrease in GP suggests the occurrence of an early conformational rearrangement preceding aggregation. A plausible explanation is a partial reorganization of the tryptophan-rich inner cavity, which may transiently alter ANS binding dynamics, reducing the accessibility of hydrophobic binding sites. The subsequent increase suggests a reorganization toward more solvent-exposed conformations, potentially involving partial unfolding and redistribution of ANS binding sites. Finally, the decrease at higher temperatures may reflect the formation of compact, aggregation-prone states or hydrophobic collapse, in which apolar regions become reburied or clustered.

These results indicate that the introduced tryptophan residues differentially affect the stability and unfolding pathway. While W4 follows a relatively simple and progressive transition, W6 undergoes a more complex sequence of conformational changes, implying the presence of metastable intermediates. Such behavior has important implications for protein stability, folding cooperativity, and potential aggregation propensity, highlighting how targeted mutations can modulate not only local environments but also global structural dynamics.

**Figure 4:**
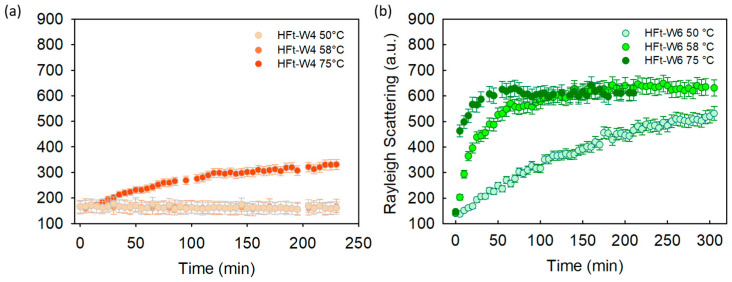
Time-dependent Rayleigh scattering intensity during isothermal incubation of 5 µM HFt-W4 (**a**) and HFt-W6 (**b**) in 20 mM sodium phosphate buffer (pH 7.4) supplemented with 150 mM NaCl at 50 °C, 58 °C, and 75 °C. Error bars represent the standard deviation.

To further elucidate the distinct aggregation behavior of HFt-W4 and HFt-W6, we performed isothermal kinetic experiments. Figure 4 shows the time-dependent evolution of Rayleigh scattering intensity for 5 µM HFt-W4 (panel a) and HFt-W6 (panel b) at 50 °C, 58 °C, and 75 °C. These temperatures were selected because they correspond to key transition regions identified in the thermal scan (Figure 2). In both variants, the increase in scattering intensity over time reflects the formation and growth of supramolecular assemblies. However, clear differences emerge between the two variants. HFt-W4 displays minimal changes at 50 °C and 58 °C, with nearly overlapping scattering profiles, suggesting negligible aggregation under these conditions. A noticeable increase is observed only at 75 °C, although the overall intensity remains significantly lower than that of HFt-W6.

In contrast, HFt-W6 shows a much more pronounced increase in scattering intensity, particularly at 58 °C and 75 °C, where the signal rises rapidly, indicating fast aggregation kinetics and efficient growth of supramolecular assemblies.

These results indicate that HFt-W6 not only aggregates to a greater extent but does so much more rapidly, especially at intermediate and high temperatures, as evidenced by the steeper rise in scattering intensity and the reduction in any apparent lag phase. Conversely, HFt-W4 appears considerably more resistant to thermally induced aggregation, requiring higher temperatures to trigger detectable assembly and still maintaining a lower overall aggregation level. This behavior underscores the markedly higher aggregation propensity and lower stability of HFt-W6 compared to HFt-W4.

**Figure 5:**
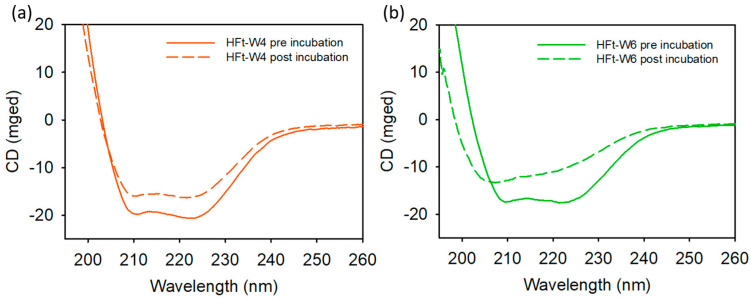
Far-UV (190–260 nm) CD spectra of 5 μM HFt-W4 (**a**) and HFt-W6 (**b**) in 20 mM sodium phosphate buffer (pH 7.4) supplemented with 150 mM NaCl, recorded before (solid line) and after (dashed line) 5 h of incubation at 75 °C. The measurements were performed in triplicate.

The structural states of aggregated HFt-W4 and HFt-W6 proteins after 5 h of incubation at 75 °C were investigated by circular dichroism (CD) spectroscopy in order to assess changes in secondary structure content upon thermal aggregation [35,36]. As shown in Figure 5, the initial spectra of both variants display the characteristic features of α-helical proteins, with two well-defined negative bands at approximately 208 and 222 nm, consistent with the native ferritin fold [9]. This confirms that, despite the introduction of additional tryptophan residues, both HFt-W4 and HFt-W6 preserve a largely α-helical structure under native conditions.

After 5 h of incubation at 75 °C, clear differences emerge between the two variants. Notably, although HFt-W4 exhibits an increase in scattering intensity under these conditions (Figure 4), its CD spectrum indicates that the secondary structure is largely preserved. HFt-W4 maintains its α-helical signature, with only a moderate decrease in ellipticity at 208 and 222 nm. This suggests that, even under aggregating conditions, the secondary structure is substantially retained. Such a mechanism is consistent with the formation of amorphous or weakly ordered aggregates that do not require extensive backbone reorganization.

In contrast, the HFt-W6 variant exhibits a pronounced variation in its CD spectrum after 5 h, as highlighted in Figure 5b. The decrease in the intensity of the α-helical bands at 208 and 222 nm is accompanied by the emergence of a single minimum around 215–218 nm, which is indicative of β-sheet-rich structures. This transition suggests a substantial conformational rearrangement during the aggregation, leading to more ordered assemblies characterized by extensive backbone interactions and intermolecular hydrogen bonding [37].

These observations point to fundamentally different aggregation pathways for the two variants. While HFt-W4 appears to aggregate through a pathway that preserves its native-like α-helical structure, HFt-W6 undergoes a structural conversion toward β-sheet–dominated assemblies. This difference may be attributed to the higher number of tryptophan residues in HFt-W6, which could enhance hydrophobic interactions and promote structural destabilization, favoring intermolecular rearrangements. Overall, the CD data indicate that the increased hydrophobic character and altered residue distribution in HFt-W6 significantly impact both the structural evolution and the final architecture of the aggregates.

## 4. Conclusions

In this work, we performed a structural characterization of two engineered human H-ferritin variants, HFt-W4 and HFt-W6, designed to modulate the hydrophobicity of the internal cavity through the introduction of four and six tryptophan residues per subunit, respectively. Expanding upon our previous findings, which established the baseline differences between these mutants and the wild-type counterpart, the thermal stability analysis presented in this work mapped the distinct conformational trajectories of the two engineered nanocages under heating stress. By combining spectroscopic and kinetic analyses over a range of temperatures, we demonstrate that subtle differences in internal hydrophobic engineering can profoundly affect protein stability, unfolding pathways, and aggregation behavior.

Rayleigh scattering experiments revealed that HFt-W6 undergoes thermally induced aggregation at significantly lower temperatures compared to HFt-W4, indicating reduced thermal stability. This observation is consistent with previous reports of impaired quaternary reassembly in HFt-W6 and suggests that excessive hydrophobicity within the cavity perturbs the delicate balance of intra- and inter-subunit interactions required for maintaining the native nanocage architecture. In contrast, HFt-W4 preserves higher thermal resistance, supporting the idea that moderate hydrophobic modification can be accommodated without compromising structural integrity.

Fluorescence measurements using the ANS probe further elucidated the role of hydrophobic interactions in these systems. The GP analysis suggested that HFt-W4 initially presents buried hydrophobic regions that become progressively exposed upon heating, following a relatively cooperative unfolding process. Conversely, HFt-W6 displays an intrinsically more solvent-accessible hydrophobic character and a complex, non-monotonic GP profile as a function of temperature, indicative of multiple conformational rearrangements and the presence of intermediate states. These findings highlight a less stable and more heterogeneous conformational landscape for HFt-W6.

Kinetic aggregation studies confirmed these differences, showing faster aggregation rates and reduced lag phases for HFt-W6, particularly at elevated temperatures. This behavior can suggest that the early exposure of hydrophobic patches promotes intermolecular interactions and nucleation events, accelerating aggregate formation.

Circular dichroism analysis provided further insight into the structural nature of the aggregates. While HFt-W4 largely retains its α-helical secondary structure even after prolonged incubation, HFt-W6 undergoes a marked transition toward β-sheet-rich conformations. 

Overall, these results suggest that increasing the number of hydrophobic residues within the ferritin cavity significantly alters the physicochemical properties of the nanocage, while simultaneously introducing structural destabilization beyond a critical threshold. These findings provide useful insights for the rational design of ferritin-based nanoplatforms, highlighting the importance of carefully balancing cavity engineering and structural stability to modulate their functional properties without compromising their overall robustness.

## Data Availability

The raw data supporting the conclusions of this article will be made available by the authors on request.
